# Visceral Kaposi's Sarcoma Presenting as Upper Gastrointestinal Bleeding

**DOI:** 10.1155/2015/438973

**Published:** 2015-05-06

**Authors:** Naomi Hauser, Devon McKenzie, Xavier Fonseca, Jose Orsini

**Affiliations:** Department of Medicine, New York University School of Medicine, Woodhull Medical and Mental Health Center, Brooklyn, NY 11206, USA

## Abstract

Since the advent of highly active antiretroviral therapy (HAART), the incidence of acquired immunodeficiency syndrome- (AIDS-) related Kaposi's sarcoma (KS) has decreased dramatically. While cutaneous KS is the most common and well-known manifestation, knowledge of alternative sites such as the gastrointestinal (GI) tract is important. GI-KS is particularly dangerous because of its potential for serious complications including perforation, obstruction, or bleeding. We report a rare case of GI-KS presenting as upper GI bleeding in a human immunodeficiency virus- (HIV-) infected transgendered individual. Prompt diagnosis and early initiation of therapy are the cornerstones for management of this potentially severe disease.

## 1. Introduction

While KS is a rare type of cancer in the United States, accounting for approximately 0.001% of all diagnosed malignancies [1], it affects HIV-infected patients at a rate of up to 1000 times greater than the general population [2]. It is the most prevalent malignancy among HIV-infected patients when HAART is not readily accessible [3, 4]. KS most commonly involves mucocutaneous sites such as the skin and oropharynx, but visceral involvement is not unusual [5, 6]. Site identification is important for prognosis and treatment. Even though HAART is seen to curb visceral involvement of KS in those with known cutaneous disease [7], the need for additional treatment, such as surgery and/or chemotherapy, may depend on the extent of visceral disease [6, 8–10]. GI tract involvement is usually asymptomatic, but when symptoms are present, they are typically nonspecific and may include abdominal pain, dyspepsia, nausea, vomiting, or diarrhea [11]. As Nagata et al. described, GI-KS can take on a variety of endoscopic appearances ranging from flat, maculopapular lesions to polypoid masses of variable sizes and consistencies [6]. Larger and more friable lesions have the potential to become severe enough to cause hemorrhage, perforation, or obstruction within the GI tract [12, 13]. We present an uncommon case of AIDS-related GI-KS with vague, nonspecific symptoms complicated by GI bleeding. Our case illustrates the need for increased awareness of the varied manifestations and complications of AIDS-related KS. A high index of suspicion along with established criteria for investigation is needed for the diagnosis and treatment of AIDS-related GI-KS, especially in cases of rapidly developing disease.

## 2. Case Report

A 35-year-old Hispanic transgendered female, born male, was brought to the emergency department (ED) complaining of cough, subjective fever, abdominal pain, nausea, and vomiting for a few days, as well as unintentional weight loss and “feeling unwell” for the previous 3 months. She reported being diagnosed with HIV infection 2 months earlier and having a negative HIV test 7 months before becoming seropositive. She was HAART-naive at the time of the ED visit. Her past medical history was significant for chronic constipation for which she used daily laxatives. On arrival to the ED, her vital signs were within normal limits. Physical examination was notable for laryngeal stridor, bilateral crackles, and coarse breath sounds, as well as for left lower quadrant tenderness to palpation. Remarkable laboratory findings included a platelet count of 74,000/*μ*L (130,000–400,000), activated partial thromboplastin time of 36.3 seconds (20.4–33.4), a sodium level of 129 mmol/L (135–147), and a potassium level of 5.5 mmol/L (3.5–5.3). Complete blood count (CBC) and liver function tests (LFTs) were within normal limits. A chest X-ray (CXR) revealed no infiltrates. The patient was discharged home with prescriptions for metoclopramide and esomeprazole.

Six days later, the patient returned to the ED complaining of blood-streaked vomit and stool, odynophagia, constipation, and generalized body pain. Vital signs were within normal limits and physical examination disclosed epigastric tenderness. Fecal occult blood test was positive and laboratory findings revealed a 3 g/dL drop in hemoglobin (Hgb) (from 12.8 g/dL to 9.7 g/dL; 12.0–16.0), a 9% drop in hematocrit (Hct) (from 35.9% to 26.9%; 37.0–47.0), worsening thrombocytopenia to 43,000/*μ*L, and a CD_4_ T-cell count of 8 cells/mm^3^ (544–1894). Coagulation profile was within normal limits. The patient was transfused one unit of packed red blood cells and admitted with a diagnosis of GI hemorrhage, likely secondary to gastroduodenal ulcer. Thorough physical examination was performed on the floor and revealed palpable red maculopapular, nonpainful, nonpruritic skin lesions on the back, thighs, and arms. At that time, the diagnosis of cutaneous KS was sought. Gastroenterology service was consulted and oral fluconazole 100 mg daily was initiated for possible* Candida* esophagitis causing the patient's odynophagia. During this admission, the patient had one episode of coffee-ground emesis without hematochezia or melena. A barium esophagram was unremarkable. HIV-1 viral load by PCR was 172,213 copies/mL and human herpesvirus- (HHV-) 8 was detected in the patient's plasma. Her clinical condition improved without further clinical evidence of GI bleeding. She was discharged on hospital day 5 on HAART,* Pneumocystis jirovecii* and* Mycobacterium avium* complex prophylaxis, and with instructions to return to HIV and GI outpatient clinics. Esophagogastroduodenoscopy (EGD) was scheduled to be performed as an outpatient.

The patient returned to the ED for a third time 2 weeks after discharge complaining of nausea, vomiting, and abdominal pain for 3 days. All vital signs were within normal limits, with the exception of a temperature of 102.5°F. Significant laboratory findings included Hgb of 8.7 g/dL, Hct of 25.4%, platelet count of 86,000/*μ*L, and sodium level of 126 mmol/L. Lactic acid level was within normal limits. Her CD_4_ T-cell count had risen to 20 cells/mm^3^ and her HIV-1 viral load had dropped to 3,530 copies/mL. Urine toxicology screen, blood cultures, cerebrospinal fluid analysis, and CXR were all negative. Urine cultures were not completed. Empiric intravenous antimicrobial therapy was initiated consisting of cefepime 1 g every 12 hours and vancomycin 1 g every 12 hours. Abdominal computed tomography (CT) with oral and intravenous contrast showed severe thickening of the antral portion of the stomach.

**Figure 1:**
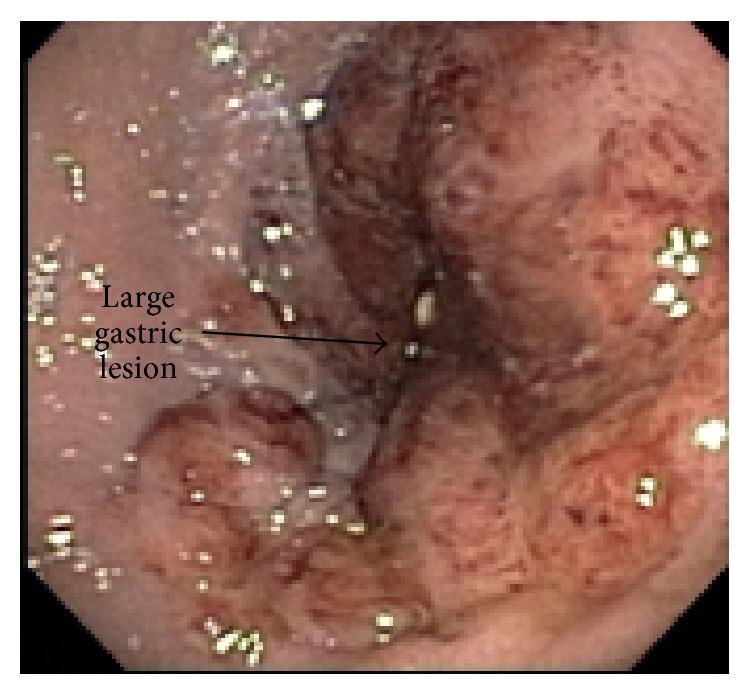
Large gastric lesion.

**Figure 2:**
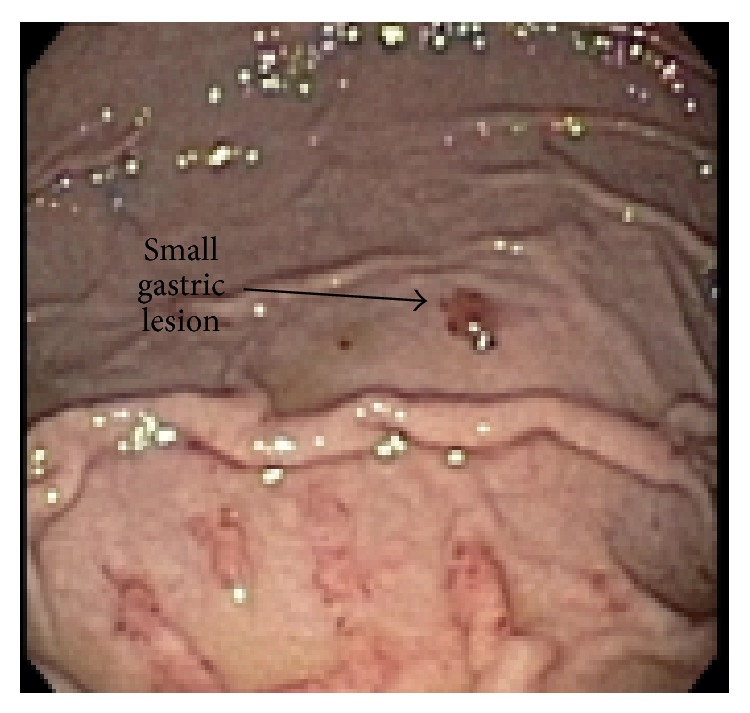
Small gastric lesion.

**Figure 3:**
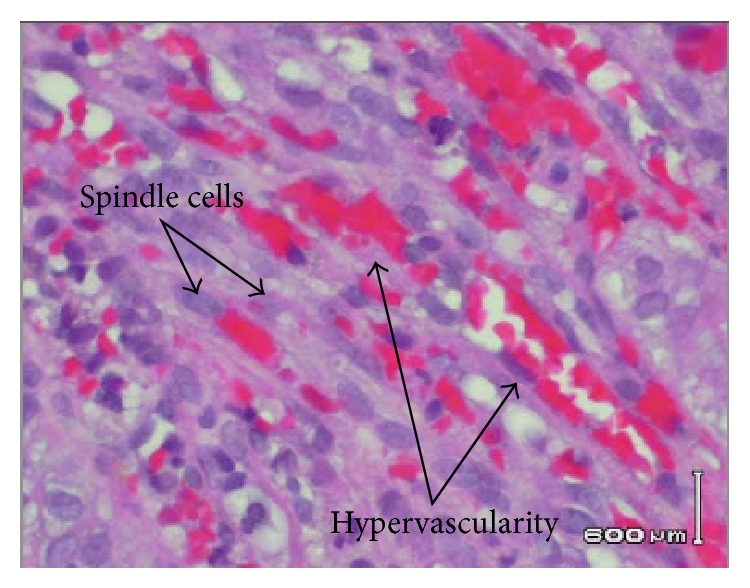
Gastric lesion biopsy displaying spindle cells and hypervascularity.

**Figure 4:**
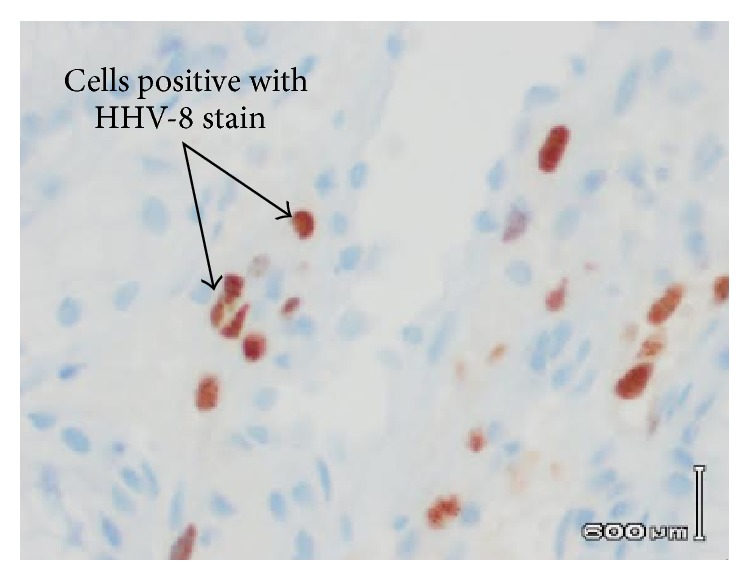
Gastric lesion biopsy showing cells positive with HHV-8 stain (brown).

EGD revealed multiple friable and possibly obstructive tumors of varying sizes in the stomach and duodenum (Figures 1 and 2). Cytopathology of the lesions showed high vascularity and spindle cells (Figure 3) and stained positively for HHV-8 (Figure 4), findings consistent with KS. The patient was discharged with instructions to continue with HAART regimen and follow up with HIV and hematology/oncology clinics. Six months after her initial ED presentation, the patient's CD_4_ count had risen to 78 cells/mm^3^ and repeat abdominal CT showed a decrease in the thickening of the gastric antrum. She began chemotherapy with doxorubicin one month later and completed four cycles before being lost for followup.

## 3. Discussion

Since the widespread adoption of HAART in the treatment of HIV infection, the incidence of KS has dramatically decreased among the HIV-infected population. Visceral involvement is often asymptomatic and frequently diagnosed postmortem [14]. Knowledge of visceral KS in general and GI-KS in particular remains important in light of the possible complications such as hemorrhage and obstruction as presented in our case. Acute GI bleeding due to GI-KS has been infrequently reported in the literature [14–16]. Neoplastic lesions are the most common causes of HIV-related upper GI bleeding, with GI-KS most frequently being asymptomatic, and gastrointestinal non-Hodgkin lymphoma often presenting with pain or gastric outlet obstruction [17]. Other GI malignancies to be considered include the much more common adenocarcinoma, which comprises nearly 95% of gastric malignancies in the general population [18]. Leiomyomas, rhabdomyosarcomas, pleomorphic sarcomas, and GI stromal tumors are additional spindle cell tumors that may be appropriate to consider as differential diagnoses [19]. Mansfield et al. analyzed 16 cases of acute upper GI bleeding associated with GI-KS reported in the literature, a handful of which were AIDS-related KS, most of the others following renal transplant and immunosuppression [14].

Nagata et al. outlined the need to determine indications for EGD in diagnosing GI-KS. They suggested that endoscopy is clearly indicated in HIV/AIDS patients with GI symptoms and cutaneous KS but that GI-KS is often present in their absence [6]. Although the mainstay of treatment is initiating/continuing HAART, early diagnosis is important in severe cases for which additional therapy is both needed and available [6, 7, 12]. Guidelines for endoscopic investigation may help prevent diagnosis of GI-KS at later stages of disease when combination therapy would no longer be tolerated [20]. Although unusual, our case illustrates the need for increased awareness of GI-KS as a potentially dangerous complication of HIV infection and as a possible etiology of upper GI bleeding. Indications for EGD in such patients should be evaluated and determined in order to prevent late diagnosis of this disease.
